# A cubic calcium oxynitrido-silicate, Ca_2.89_Si_2_N_1.76_O_4.24_

**DOI:** 10.1107/S1600536811042607

**Published:** 2011-10-29

**Authors:** Ali Sharafat, Pedro Berastegui, Saeid Esmaeilzadeh, Lars Eriksson, Jekabs Grins

**Affiliations:** a School of Engineering, Linné University, Växjö, S-351 95 Växjö, Sweden; b Department of Materials and Environmental Chemistry, Arrhenius Laboratory, Stockholm University, S-106 91 Stockholm, Sweden

## Abstract

The title compound, tricalcium oxynitride silicate, with composition Ca_3-*x*_Si_2_N_2-2*x*_O_4+2*x*_ (*x* ≃ 0.12), is a perovskite-related calcium oxynitrido silicate containing isolated oxynitrido silicate 12-rings. The N atoms are statistically disordered with O atoms (occupancy ratio N:O = 0.88:0.12) and occupy the bridging positions in the 12 ring oxynitrido silicate anion, while the remaining O atoms are located at the terminal positions of the Si(O,N)_4_ tetrahedra. The majority of the Ca^2+^ cations fill the channels along [100] in the packing of the 12-ring anions. The rest of these cations are located at several positions, with partial occupancy, in channels along the body diagonals.

## Related literature

For a closely related silicate, as well as a germanate, see: Fischer & Tillmanns (1984 ▶) and for a more distantly related calcium aluminate, see: Mondal & Jeffery (1975 ▶)

## Experimental

### 

#### Crystal data

Ca_2.89_Si_2_N_1.76_O_4.24_
*M* *_r_* = 264.65
Cubic, 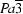

*a* = 15.0626 (1) Å
*V* = 3417.45 (4) Å^3^
*Z* = 24
Mo *K*α radiation
μ = 3.18 mm^−1^
*T* = 293 K
0.10 × 0.06 × 0.02 mm

#### Data collection

Oxford Diffraction XcaliburIII with Sapphire-3 CCD diffractometer
Absorption correction: multi-scan (*CrysAlis RED*; Oxford Diffraction, 2008 ▶) *T*_min_ = 0.67, *T*_max_ = 0.94
30793 measured reflections
1982 independent reflections
1779 reflections with *I* > 2s(*I*)
*R*_int_ = 0.027

#### Refinement

*R*[*F* ^2^ > 2σ(*F* ^2^)] = 0.032
*wR*(*F* ^2^) = 0.069
*S* = 1.22
1982 reflections
113 parameters
Δρ_max_ = 0.87 e Å^−3^
Δρ_min_ = −0.76 e Å^−3^

### 

Data collection: *CrysAlis CCD* (Oxford Diffraction, 2008 ▶); cell refinement: *CrysAlis RED* (Oxford Diffraction, 2008 ▶); data reduction: *CrysAlis RED*; program(s) used to solve structure: *SHELXS97* (Sheldrick, 2008 ▶); program(s) used to refine structure: *SHELXL97* (Sheldrick, 2008 ▶); molecular graphics: *DIAMOND* (Brandenburg, 2001 ▶); software used to prepare material for publication: *PLATON* (Spek, 2009 ▶) and *SHELXL97*.

## Supplementary Material

Crystal structure: contains datablock(s) I, global. DOI: 10.1107/S1600536811042607/fj2455sup1.cif

Structure factors: contains datablock(s) I. DOI: 10.1107/S1600536811042607/fj2455Isup2.hkl

Additional supplementary materials:  crystallographic information; 3D view; checkCIF report

## Figures and Tables

**Table 1:** Selected bond lengths (Å)

Si1—O4	1.6245 (15)
Si1—O3	1.6476 (14)
Si1—N2/O2	1.6788 (18)
Si1—N1/O1	1.6958 (17)
Si2—O6	1.6341 (15)
Si2—O5	1.6485 (15)
Si2—N1/O1^i^	1.6899 (17)
Si2—N2/O2	1.6942 (18)

Symmetry code: (i) 
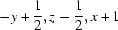
.

**Fig. 1.**
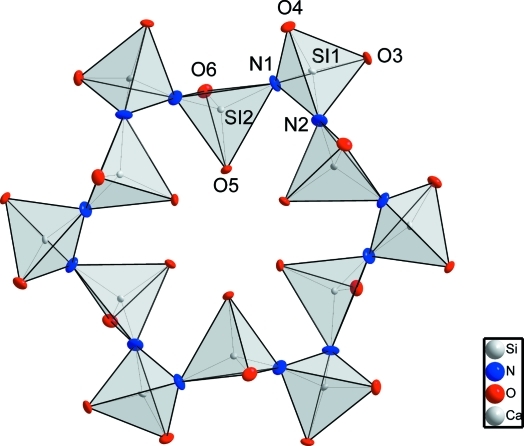
The oxynitrido-silicate anion of the title compound (I) with the unique atoms of the oxynitrido-silicate anion labelled. Displacement ellipsoids drawn at 50% probability.

**Fig. 2.**
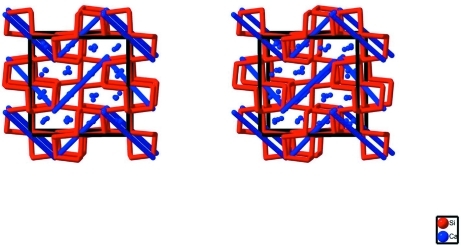
Stereoview of the unitcell viewed approximately along [100] with the oxynitrido-silicate anions marked with red colour and the Ca atoms ffwith blue colour. The disordered arrays of Ca atoms along the <111> shown by a solid rod.

## supplementary crystallographic information

### Comment

The title compound is a crystalline component formed in the melts of oxynitrido glasses, studied at our department. The title compound calcium oxynitrido-silicate contains isolated 12-ring anions with the ideal composition Ca_3 -_*_x_*Si_2_N_2–2x_O_4 + 2x_ where the title compound have *x*≈ 0.12. It should be emphasized that *x*=0 do not indicate an end member of a possible solid solution series of compounds. The nitrogen atoms in the anions occupies mainly the ring positions in the 12 ring while the oxygen atoms mainly occupy the apex positions. A figure of the oxynitrido-silicate anion is shown in Fig. 1. In Fig. 2 a simplified packing is shown where the arrangement of Ca atoims in channels along <100> as well as the disordered arrangement along the <111> directions. 56 Ca atoms in each unit cell fills channels along <100> in the packing of the 12-ring anions while the rest of the Ca positions are located in channels along <111> and show tendencies to be disordered. The split positions of the Ca cations along the <111> can be viewed as a consequence of the implied Pa-3 symmetry. Whether the space group should better be *P*2_1_3 or even *P*2_1_2_1_2_1_ with cubic twinning is unfortunately not possible to determine, neither from systematic reflection conditions nor from investigations of the s.u. of the cell parameters. No One can refine orthorhombic unit-cell parameters but if one should beleive the e.s.d.'s is more of an open question. We choose to describe the structure with highest possible symmetry but at the price of some disorder. Similar arrangements of cations and ring formed anions are found in the structurally related compounds K_4_SrGe_3_O_9_ and Na_4_CaSi_3_O_9_ (Fischer & Tillmanns, 1984).

### Experimental

The title compound was obtained by slow cooling of a melt with the nominal composition Ca_1.71_Si_2_O_1.71_N_2.67_ from 1700° C by 1° C / min in a graphite furnace.

### Refinement

In order to match the refined Ca composition the N1 and N2 position were mixed occupied by 88° N and 12°O. Attempts to refine the N/O ratio from the X-ray diffraction data failed using the present single-crystal data as a consequence of the close resemblance of the atomic form factors of N and O. The occupancy factors of the Calcium ions did converge to the composition reported in the title and was fully consistent with results from EDS analyses giving the ratio Ca/Si = 0.59 (1). The refined Ca content 2.88 and 2 Si give the Ca/Si = 0.59. It must be emphasized that the precise occupation of Ca atoms are heavily dependent on the precise model used. Including a larger number of Ca atoms, one could refine the model slightly closer to the ideal composition Ca_3_Si_2_O_4_N_2_.

### Crystal data

**Table:**

Ca_2.89_Si_2_N_1.76_O_4.24_	*D*_x_ = 3.087 Mg m^−^^3^
*M**_r_* = 264.65	Mo *K*α radiation, λ = 0.71073 Å
Cubic, *P**a*3	Cell parameters from 19622 reflections
Hall symbol: -P 2ac 2ab 3	θ = 3.8–32.2°
*a* = 15.0626 (1) Å	µ = 3.18 mm^−^^1^
*V* = 3417.45 (4) Å^3^	*T* = 293 K
*Z* = 24	Block, colourless
*F*(000) = 3158	0.10 × 0.06 × 0.02 mm

### Data collection

**Table:**

Oxford Diffraction XcaliburIII with Sapphire-3 CCD diffractometer	1982 independent reflections
Radiation source: fine-focus sealed tube	1779 reflections with *I* > 2s(*I*)
graphite	*R*_int_ = 0.027
Detector resolution: 16.5467 pixels mm^-1^	θ_max_ = 32.3°, θ_min_ = 3.8°
ω scans at different φ	*h* = −22→18
Absorption correction: multi-scan (*CrysAlis RED*; Oxford Diffraction, 2008)	*k* = −18→21
*T*_min_ = 0.67, *T*_max_ = 0.94	*l* = −22→21
30793 measured reflections	

### Refinement

**Table:**

Refinement on *F*^2^	Primary atom site location: structure-invariant direct methods
Least-squares matrix: full	Secondary atom site location: difference Fourier map
*R*[*F*^2^ > 2σ(*F*^2^)] = 0.032	*w* = 1/[σ^2^(*F*_o_^2^) + (0.026*P*)^2^ + 5.7834*P*] where *P* = (*F*_o_^2^ + 2*F*_c_^2^)/3
*wR*(*F*^2^) = 0.069	(Δ/σ)_max_ = 0.001
*S* = 1.22	Δρ_max_ = 0.87 e Å^−^^3^
1982 reflections	Δρ_min_ = −0.76 e Å^−^^3^
113 parameters	Extinction correction: *SHELXL97* (Sheldrick, 2008), Fc^*^=kFc[1+0.001xFc^2^λ^3^/sin(2θ)]^-1/4^
0 restraints	Extinction coefficient: 0.00048 (5)

### Special details

**Table:**

Geometry. All e.s.d.'s (except the e.s.d. in the dihedral angle between two l.s. planes) are estimated using the full covariance matrix. The cell e.s.d.'s are taken into account individually in the estimation of e.s.d.'s in distances, angles and torsion angles; correlations between e.s.d.'s in cell parameters are only used when they are defined by crystal symmetry. An approximate (isotropic) treatment of cell e.s.d.'s is used for estimating e.s.d.'s involving l.s. planes.
Refinement. Refinement of *F*^2^ against ALL reflections. The weighted *R*-factor *wR* and goodness of fit *S* are based on *F*^2^, conventional *R*-factors *R* are based on *F*, with *F* set to zero for negative *F*^2^. The threshold expression of *F*^2^ > σ(*F*^2^) is used only for calculating *R*-factors(gt) *etc*. and is not relevant to the choice of reflections for refinement. *R*-factors based on *F*^2^ are statistically about twice as large as those based on *F*, and *R*- factors based on ALL data will be even larger.

### Fractional atomic coordinates and isotropic or equivalent isotropic displacement parameters (Å^2^)

**Table:**

	*x*	*y*	*z*	*U*_iso_*/*U*_eq_	Occ. (<1)
Si1	0.00530 (3)	0.27221 (3)	0.76315 (3)	0.00633 (9)	
Si2	0.01658 (3)	0.24358 (3)	0.98439 (3)	0.00567 (9)	
N1	0.01861 (12)	0.37791 (11)	0.72676 (13)	0.0176 (3)	0.88
O1	0.01861 (12)	0.37791 (11)	0.72676 (13)	0.0176 (3)	0.12
N2	0.00891 (13)	0.26556 (13)	0.87435 (11)	0.0193 (4)	0.88
O2	0.00891 (13)	0.26556 (13)	0.87435 (11)	0.0193 (4)	0.12
O3	0.09048 (10)	0.21494 (9)	0.72534 (9)	0.0131 (3)	
O4	−0.09065 (10)	0.23612 (10)	0.72964 (10)	0.0160 (3)	
O5	−0.01845 (10)	0.33802 (10)	1.02719 (10)	0.0147 (3)	
O6	−0.04423 (10)	0.15759 (10)	1.01040 (10)	0.0153 (3)	
Ca1	−0.12893 (2)	0.37107 (2)	1.12893 (2)	0.01028 (12)	
Ca2	−0.11371 (3)	0.38333 (3)	0.90382 (3)	0.01162 (8)	
Ca3	0.13964 (3)	0.13071 (3)	0.85405 (3)	0.01601 (9)	
Ca4	0.24330 (14)	0.25670 (14)	0.74330 (14)	0.0094 (6)*	0.1715 (6)
Ca5	0.1757 (2)	0.3243 (2)	0.6757 (2)	0.0094 (2)*	0.140 (2)
Ca6	0.15523 (5)	0.34477 (5)	0.65523 (5)	0.0094 (2)*	0.670 (3)
Ca7	0.1254 (9)	0.3746 (9)	0.6254 (9)	0.0094 (2)*	0.043 (3)
Ca8	0.1031 (7)	0.3969 (7)	0.6031 (7)	0.0094 (2)*	0.076 (2)
Ca9	0.0819 (5)	0.4181 (5)	0.5819 (5)	0.0094 (2)*	0.086 (3)
Ca10	0.0572 (3)	0.4428 (3)	0.5572 (3)	0.0094 (2)*	0.099 (2)
Ca11	0.0166 (4)	0.4834 (4)	0.5166 (4)	0.0094 (2)*	0.095 (3)
Ca12	0.0000	0.5000	0.5000	0.0094 (2)*	0.603 (6)

### Atomic displacement parameters (Å^2^)

**Table:**

	*U*^11^	*U*^22^	*U*^33^	*U*^12^	*U*^13^	*U*^23^
Si1	0.0077 (2)	0.0061 (2)	0.0052 (2)	−0.00013 (16)	−0.00028 (15)	−0.00047 (15)
Si2	0.00564 (19)	0.0064 (2)	0.0049 (2)	0.00030 (16)	0.00068 (15)	−0.00020 (16)
N1	0.0206 (8)	0.0063 (7)	0.0260 (9)	0.0011 (6)	0.0043 (7)	0.0004 (6)
O1	0.0206 (8)	0.0063 (7)	0.0260 (9)	0.0011 (6)	0.0043 (7)	0.0004 (6)
N2	0.0240 (9)	0.0274 (9)	0.0066 (7)	−0.0007 (7)	0.0010 (6)	−0.0004 (6)
O2	0.0240 (9)	0.0274 (9)	0.0066 (7)	−0.0007 (7)	0.0010 (6)	−0.0004 (6)
O3	0.0149 (6)	0.0105 (6)	0.0140 (6)	0.0035 (5)	0.0042 (5)	−0.0008 (5)
O4	0.0150 (6)	0.0162 (7)	0.0169 (7)	−0.0055 (5)	−0.0065 (5)	0.0014 (5)
O5	0.0174 (7)	0.0124 (6)	0.0144 (6)	0.0057 (5)	−0.0035 (5)	−0.0062 (5)
O6	0.0157 (7)	0.0132 (6)	0.0170 (7)	−0.0063 (5)	0.0040 (5)	0.0008 (5)
Ca1	0.01028 (12)	0.01028 (12)	0.01028 (12)	−0.00048 (12)	0.00048 (12)	0.00048 (12)
Ca2	0.01262 (17)	0.00917 (16)	0.01306 (17)	0.00006 (12)	−0.00140 (13)	0.00004 (12)
Ca3	0.01781 (19)	0.01586 (19)	0.01435 (18)	0.00563 (14)	−0.00307 (14)	−0.00105 (14)

### Geometric parameters (Å, °)

**Table:**

Si1—O4	1.6245 (15)	Si2—O6	1.6341 (15)
Si1—O3	1.6476 (14)	Si2—O5	1.6485 (15)
Si1—N2	1.6788 (18)	Si2—N1^i^	1.6899 (17)
Si1—N1	1.6958 (17)	Si2—N2	1.6942 (18)
			
O4—Si1—O3	114.22 (8)	O6—Si2—N1^i^	109.58 (9)
O4—Si1—N2	108.60 (9)	O5—Si2—N1^i^	108.13 (9)
O3—Si1—N2	106.76 (9)	O6—Si2—N2	110.56 (9)
O4—Si1—N1	108.59 (9)	O5—Si2—N2	101.08 (9)
O3—Si1—N1	106.72 (8)	O1^i^—Si2—N2	113.07 (9)
N2—Si1—N1	112.00 (10)	N1^i^—Si2—N2	113.07 (9)
O6—Si2—O5	114.26 (8)	Si2^ii^—N1—Si1	142.98 (12)
O6—Si2—O1^i^	109.58 (9)	Si1—N2—Si2	171.87 (14)
O5—Si2—O1^i^	108.13 (9)		
			
N2—Si1—N1—Si2^ii^	−46.7 (2)		

Symmetry codes: (i) −*y*+1/2, *z*−1/2, *x*+1; (ii) *z*−1, −*x*+1/2, *y*+1/2.
